# ER and COX2 expression in endometrial hyperplasia processes

**DOI:** 10.1097/MD.0000000000034864

**Published:** 2023-08-18

**Authors:** Nataliia Tsyndrenko, Mykola Lyndіn, Kateryna Sikora, Andrew Awuah Wireko, Toufik Abdul-Rahman, Nataliia Hyriavenko, Anatolii Romaniuk

**Affiliations:** a Sumy State University, Sumy, Ukraine; b Institute of Anatomy, Medical Faculty, University of Duisburg-Essen, Essen, Germany.

**Keywords:** cyclooxygenase-2, endometrial hyperplasia, endometrial polyps, estrogen receptor, hyperplastic endometrial processes, immunohistochemistry

## Abstract

Endometrial hyperplastic processes (EHPs) encompass various morphological changes, characterized by an increased ratio of endometrial glands to stroma. These changes manifest as endometrial hyperplasia (EH) and endometrial polyps. The objective of this study was to investigate the expressions of ER and Cyclooxygenase-2 (COX2) in EH and endometrial polyps, and determine their correlation with histological and anthropometric parameters. Tissue samples were obtained during hysteroresectoscopy and divided into 3 groups: non-atypical EH, glandular EP, and glandular-fibrous EP. We examined the immunoprofile of epithelial and stromal cells using rabbit polyclonal anti-COX2 antibodies and rabbit monoclonal anti-ER antibodies (clone SP1). Our results indicate that there is no association between the expressions of ER and COX2 and the type of EHP. Furthermore, the expression levels of ER and COX2 are not influenced by the patients anthropometric parameters. However, tissues with EHPs exhibited significantly higher COX2 expression compared to intact tissues. We also observed a direct correlation between ER and COX2 expression in the endometrial epithelium. The variability in ER and COX2 expressions observed in hyperplastic processes of the endometrium potentially suggests their synergistic involvement in the initiation and progression of EHPs, as well as their potential role in subsequent tumor transformation.

## 1. Introduction

Endometrial hyperplastic processes (EHPs) encompass various morphological changes in the endometrium, characterized by an increased ratio of endometrial glands to stroma compared to normal proliferative endometrium.^[1]^ EHPs are represented by endometrial hyperplasia (EH) and endometrial polyps (EPs), which can progress to endometrial cancer. The malignancy rate depends on factors such as the degree of architectural distortion and the presence of nuclear atypia.^[2]^

EH shows a non-physiological and noninvasive proliferation of the endometrium.^[3]^ It can be a polyclonal proliferative pathological process or a monoclonal precancerous lesion differentiated based on cytological atypia.^[4]^ The risk of EH progression to endometrioid adenocarcinomas ranges from 1.0% (for EH without atypia) to 46.2% (for atypical EH).^[5]^ EPs are morphologically characterized by localized hyperplastic growth of the stroma and endometrial glands and have a prevalence of 7.8% to 34.9% in different populations.^[6]^ EPs contain thick-walled blood vessels and are lined with pseudostratified or squamous epithelium.^[7]^ The rate of EP malignancy ranges from 0.8% to 8%.^[8]^

Chronic hyperestrogenism is the most important risk factor for EHP development.^[9]^ Estrogen interacts with estrogen receptors (ER) and induces endometrial cell proliferation during the proliferative stage. In the endometrium, adequate expression of ERs, including ERα, ERβ, and the G-protein-coupled ER, is critical for a regular menstrual cycle and possible pregnancy. Their impaired expression can cause many diseases, such as endometriosis, EHP, and endometrial carcinomas. The interaction of estrogen with ERα promotes the proliferation of uterine cells, and their imbalance is closely associated with an increased risk of endometrial carcinomas. In addition, the ERβ expression level has the opposite effect on ERα function.^[10]^ To some extent, ER expression predicts response to conservative treatment with progestin in women with EHP.^[11]^ Lower ER expression correlates with increased malignant transformation of EHP.^[1,7,12–14]^

Cyclooxygenase-2 (COX2) catalyzes the biosynthesis of prostaglandins from arachidonic acid. It is expressed in normal tissues and in many cancers and performs a pleiotropic and multifaceted function in carcinogenesis.^[15–17]^ COX2 is involved in regulates resistance to apoptosis, cancer cell proliferation and invasion, angiogenesis, inflammation, and metastasis. Inhibition of the activity of these proteins may contribute to the regression of a malignant tumor, which is a promising therapeutic method in modern oncology.^[18]^

COX2 is an early response gene that can be induced by oncogenes, tumor promoters, and carcinogens.^[19]^ There is a correlation between chronic inflammation and the development of malignant tumors, contributing to the development of > 15% of carcinomas worldwide.^[20,21]^ COX2 is a potential marker of the neoplastic transformation of normal cells into a tumor.^[17,20,22,23]^ COX2-induced prostaglandin E2 production initiates a signaling cascade leading to the intensification of various metabolic processes and apoptosis inhibition or prevention. The COX2 expression level can serve as a prognostic marker for targeted treatment and indicate the degree of endometrial tumor invasion into the myometrium.^[7]^ Along with the pro-oncogenic properties of COX2, there is evidence of its tumor-suppressive effect. COX2-induced synthesis of 8-hydroxyoctanoic acid can inhibit tumor growth and cell migration.^[23]^

In this study, we evaluated the correlation between EHP histological variants [non-atypical EH, glandular endometrial polyps (GEP), and glandular-fibrous endometrial polyps (GFEP)] and anthropometric parameters in women. We also established ER and COX2 expression in different histological variants of EHP and their correlation dependence.

## 2. Materials and methods

This study used EHP tissue samples obtained after surgical treatment (hysteroresectoscopy) at the Sumy Regional Clinical Oncological Dispensary (Sumy, Ukraine) between 2020 and 2022. All patients signed the written informed consent for histological examination and personal data processing.

All cases were divided into 3 groups depending on the results of their histological examination: group I comprised non-atypical EH samples, group II comprised GEP samples, and group III comprised GFEP samples. Histological samples obtained from women receiving nonsteroidal anti-inflammatory drugs, hormones, and antihormonal drugs were excluded from this study.

We evaluated the anthropometric parameters of women (age, height, body weight, and body mass index [BMI]) and the correlation of these parameters with EHP type. BMI was calculated using the formula: BMI = m/h^2^, where m represents body weight (kg), and h represents body height (m).

The tissue samples were obtained during hysteroresectoscopy. Diagnostic hysteroscopy was performed using a Karl Storz BETTOCCHI office hysteroscope (Germany), 6 mm in diameter, with a 30° viewing angle; a 0.9% NaCl solution was used as the optical medium. Hysteroresectoscopy was performed using a resectoscope with a diameter of 9 mm and a HOPKINS telescope with a diameter of 4 mm and a viewing angle of 30°; Turusol optical medium was used in this case. The obtained tissue fragments were fixed in a buffered formalin solution, dehydrated, and embedded in paraffin.

Immunohistochemistry (IHC) and protein visualization were performed according to the manufacturer’s recommendations for the detection system used as described previously.^[17]^ Receptor imaging was conducted using the Ultra Vision Quanto Detection System HRP DAB Chromogen (Thermo Scientific, USA), which involves blocking endogenous peroxidase activity with hydrogen peroxide (10 minutes), conjugation with primary antibodies (30 minutes), blocking nonspecific background signals using Ultra V Block (5 minutes), and amplifying the reaction with Primary Antibody Amplifier Quanto (10 minutes). The final visualization was performed with diaminobenzidine under a microscope (the expression patterns were brown). Cell visualization was performed using Mayer’s hematoxylin. This study used rabbit polyclonal anti-COX2 antibodies (Diagnostic Biosystems; Pleasanton, CA) and rabbit monoclonal anti-ER antibodies (clone SP1).

The ER expression results were evaluated using the H-Score scale. Here, we used the formula: S = 1a + 2b + 3c, where a represents weakly stained cell nuclei (%), b represents moderately stained cell nuclei (%), and c represents strongly stained cell nuclei (%). Reactions were categorized based on their S value: 0 to 10, negative; 11 to 100, weakly positive; 101 to 200, moderately positive; 201 to 300, strongly positive.

The COX2 expression results were evaluated using a combined method based on the percentage of stained cells and the staining intensity. The percentage of stained cells was categorized as follows: 0, 0% stained cells; 1, 1% to 25% of stained cells; 2, 26% to 50% of stained cells; 3, > 50% stained cells. The staining intensity was graded as follows: 0, negative; 1, weakly positive; 2, moderately positive; 3, strongly positive. The obtained results were summed. Both score sets were multiplied to create a composite score: −, 0 points; +, 1 to 2 points; ++, 3 to 4 points; +++, 5 to 6 points.

All examinations were performed using a Carl Zeiss Primo Star microscope with a Zeiss AxioCam ERс 5s digital camera and the ZEN 2 (blue edition) software (Oberkochen, Germany). Data processing was performed with IBM SPSS Statistics for Windows, version 29.0 (IBM Corp., Armonk, NY) Statistics 29.0 for Windows. We calculated the arithmetic mean (M) and standard deviation, and the data are presented as M ± standard deviation. Differences between comparable indicators were assessed using Mann-Whitney (U) tests. The significance of differences between the 3 groups was assessed with a 1-way ANOVA analysis. Correlations between the indicators were evaluated with the nonparametric Spearman’s rank correlation coefficient (r). Results with *P* < .05 (95% confidence) were considered statistically significant.

## 3. Results

### 3.1. Patient group characteristics

The average age of women with EHP was 47.22 ± 10.49 years (Table 1). In addition, women with GEP (37.86 ± 5.6 years) were slightly younger (*F* = 4.19, *P* = .021) than women with EH (50.87 ± 9.3 years) or GFEP (47.59 ± 10.84 years). The average height of the women was 164.26 ± 4.85 cm and barely differed between the 3 studied groups (*F* = 0.009, *P* = .99). The average weight of the women was 73.24 ± 16.4 kg. While women with GEP appeared to generally have a lower body weight (60.57 ± 5.2 kg) than women in the other 2 groups (77.4 ± 16.1 and 74.1 ± 17.1 kg), the difference was not significant (*F* = 2.82, *P* = .07). The average BMI of all the women was 27.18 ± 6.2. Notably, 51% of women were overweight or obese (various degrees of obesity). However, we did not observe a significant difference in BMI among the groups (*F* = 2.67, *P* = .08).

**Table 1:** Patient group characteristics.

	Age	Weight	Height	BMI	ER, epithelium %	ER, stroma %	COX2
EHP	47.22 ± 10.49*	73.24 ± 16.4	164,26 ± 4,9	27,18 ± 6,2	227,2 ± 58,2	164,5 ± 51,9	4,22 ± 1,11
EH	50.87 ± 9,3*	77,4 ± 16,1	164,13 ± 4,8	28,55 ± 4,9	216 ± 75,7	186,7 ± 51,5	3,67 ± 1,03
GEP	37,86 ± 5,6*	60.57 ± 5,2	164,43 ± 4,9	22,4 ± 1,6	245,7 ± 38,7	165,7 ± 47,9	4,17 ± 1,17
GFEP	47,59 ± 10.8*	74.1 ± 17.1	164,28 ± 5,1	27,62 ± 7,1	228,5 ± 52	152,8 ± 50.9	4,83 ± 0.98

BMI = body mass index, EH = endometrial hyperplasia, EHP = endometrial hyperplastic process, ER = estrogen receptors, GEP = glandular endometrial polyps, GFEP = glandular-fibrous endometrial polyps.

There was a significant difference between patient groups – * *P* < .05.

We compared the results obtained for all EHP cases and observed a direct correlation between the women’s age and their weight (*R* = 0.72, *P* < .01) and BMI (*R* = 0.71, *P* < .01). However, separate analyses in each group showed that these correlations existed only in women with GFEP (*R* = 0.78 with *P* < .01 and *R* = 0.75 with *P* < .01, respectively); this correlation was not detected in the other 2 groups (*Р* ˃ .05).

## 4. Histological characteristics of EHP samples

**Figure 1.**
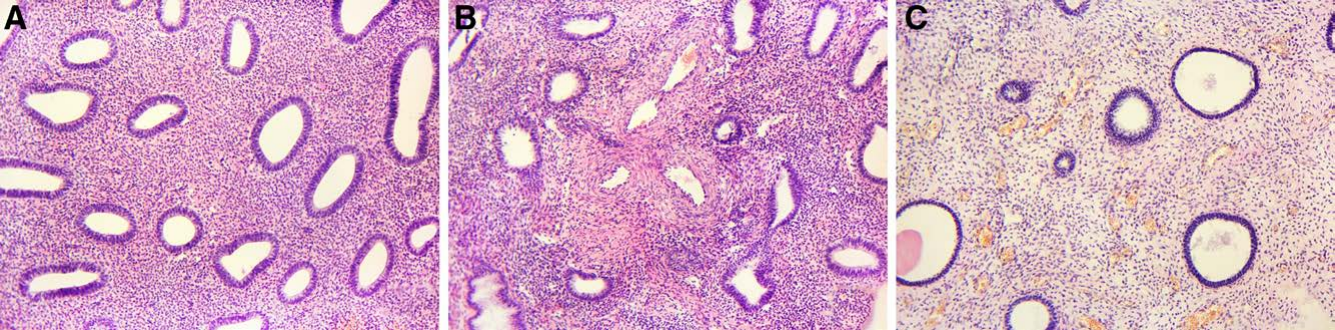
Examples of EHP. (А) EH, (В) GEP, (С) GFEP. Hematoxylin and eosin staining. Magnification × 200. EH = endometrial hyperplasia, GEP = glandular endometrial polyps, GFEP = glandular-fibrous endometrial polyps.

Non-atypical EH showed numerous irregularly distributed glands of different shapes and sizes in the uterine mucosa. Adenomatous EH showed densely located branched glands with folds toward the glands’ lumen. In some cases, cystic dilatations were present. Glandular epithelium almost did not differ from the endometrial glands epithelium in the proliferation stage. It was mostly 1-, 2-, or 3-rowed. The cells had oval nuclei and basophilic cytoplasm. Mitoses were observed in individual cells (≤ 5 in 10 fields of view). The endometrium’s stroma was predominantly cytogenic, with many cells having oval nuclei (fibroblast-like cells) and poor cytoplasm. In addition, diffuse lymphocytic infiltration and focal edema were noted in the stroma (Fig. 1A).

GEPs were formed from the endometrium’s basal layer and were represented by a localized overgrowth of glands and stroma. They appeared as unevenly distributed glands of various shapes and sizes with mostly dense stroma. The glands were lined with indifferent or proliferative-type epithelium. Glandular hyperplasia foci were observed in individual areas. Usually, a cluster of vessels with thickened walls was located at the base of a polyp (Fig. 1B). The GFEP samples were characterized by pronounced fibrotization of the stroma and fewer glands. In addition, the epithelium lining the glands’ lumen was mainly 1- or 2-rowed (Fig. 1C). Similar to EH, areas of focal lymphocytic infiltration were observed in polyp tissues.

In 26% of cases, there were multiple EPs (several polyps were simultaneously present in the uterine cavity). It should be noted that EH and polyp cases with cellular atypia were excluded from this study.

### 4.1. IHC of ER and COX2 expression

ER expression was detected in all the studied samples, both in the endometrial glands epithelium and the stroma cells. The ER expression had an inhomogeneous pattern (both among individual glands and within the same gland) represented by the interchange of receptor-positive and receptor-negative cells. In some cases, we observed the focal location of ER-positive and ER-negative epithelial and stromal cells.

**Figure 2.**
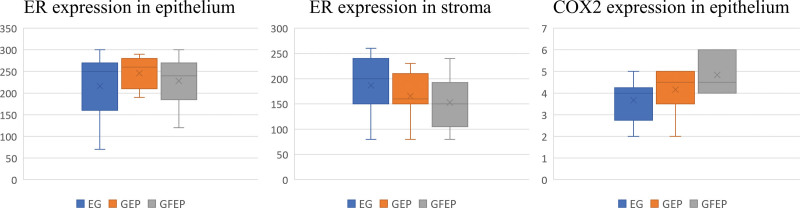
Comparative characteristics of ER and COX2 expression in EH, GEP, and GFEP histological samples. COX2 = Cyclooxygenase-2, EH = endometrial hyperplasia, ER = estrogen receptors, GEP = glandular endometrial polyps, GFEP = glandular-fibrous endometrial polyps.

**Figure 3.**
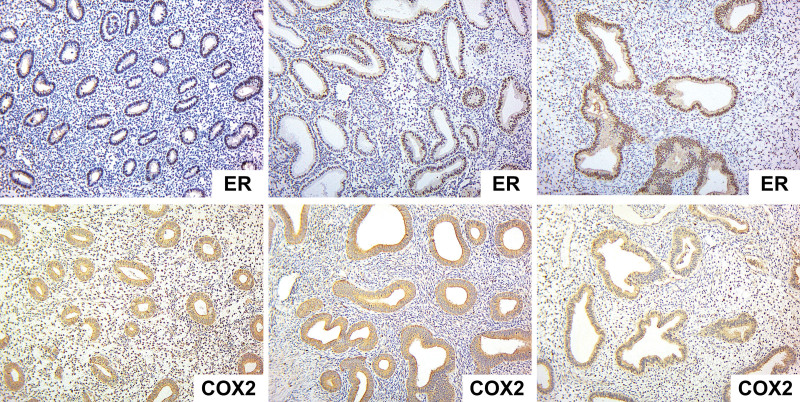
Several cases of EHP tissue samples with strong expression of ER and COX2. Magnification × 200. COX2 = Cyclooxygenase-2, ER = estrogen receptors.

In the H-score assessment of the extent of nuclear immunoreactivity for ER, the endometrial glands epithelium showed strong staining (200–300 points) in 72.5%, moderate staining (100–200 points) in 23.5%, and weak staining (0–100 points) in 4% of all EHP cases. The group-specific distributions were as follows: EH – 67% strong, 20% moderate, and 13% weak staining; GEP – 86% strong and 14% moderate staining; GFEP – 72% strong and 28% moderate staining. Generally, the immunoreactivity of epithelial cells was 216 ± 75.7 in EH samples, 245.7 ± 38.7 in GEP samples, and 228.5 ± 52 in GFEP samples (Table 1; Figs. 2 and 3). ER expression in the epithelium did not differ significantly between the groups with different histological EHP variants (*F* = 0.63, *P* = .54).

The ER expression in the EHP stroma showed strong staining in 31% of cases, moderate staining in 57%, and weak staining in 12%. The group-specific distributions were as follows: EH – 53% strong, 40% moderate, and 7% weak staining; GEP – 29% strong, 57% moderate, and 14% weak staining; GFEP – 21% strong, 65% moderate, and 14% weak staining. Generally, the immunoreactivity of stromal cells was 186.7 ± 51.5 in EH samples, 165.7 ± 47.9 in GEP samples, and 152.8 ± 50.9 in GFEP samples (Table 1, Figs 2 and 3). ER expression in stromal cells did not differ significantly among groups (*F* = 2.21, *P* = .12). However, we generally detected significantly lower ER expression in the stroma in both EHP samples and in each group (*P* < .05).

COX2 expression was detected in the epithelium of all EHP tissue samples. It was found mainly in the apical part of the prismatic epithelium’s cytoplasm. Some COX2-positive lymphocytes were found in the endometrial stroma. The average expression in the endometrial epithelium was 4.22 ± 1.11 points. It amounted to 3.67 ± 1.03 points in EH samples, 4.17 ± 1.17 points in GEP samples, and 4.83 ± 0.98 points in GFEP samples. We did not observe a significant difference in COX2 expression among the 3 groups of EHP tissue samples (*F* = 1.81, *P* = .197). However, their manifestation significantly exceeded the values of intact endometrial tissue that showed only focal COX2 expression in the luminal and glandular epithelial cells regardless of the menstrual cycle phase.^[17]^

ER and COX2 expression in endometrial tissues was not correlated with age, weight, and BMI (*Р* ˃ .05; Table 2). However, COX2 expression was inversely correlated with height in EHP samples in general (*r* = −0.49, *P* = .037) and in GFEP samples in particular (*r* = −0.85, *P* = .034). In addition, there was a direct correlation between COX2 and ER expression in the endometrial glands’ epithelium in GFEP samples (*R* = 0.91, *P* = .013). Moreover, there was a direct correlation between ER expression in the endometrium’s epithelium and stroma in EHP samples in general (*R* = 0.49, *P* < .01) and in GFEP samples in particular (*R* = 0.55, *P* < .01).

**Table 2:** Parameters of correlation between ER and СОХ-2 expression and anthropometric parameters in women.

		Age	Weight	Height	BMI	ER, epithelium	ER, stroma	COX2
EHP	ER, epithelium	*R* = 0.23, *P* = .1	r = −0.11, *P* = .43	r = −0.01, *P* = .99	r = −0.01, *P* = .94		*R* = 0.49, *P* < .01	*R* = 0.45, *P* = .06
	ER, stroma	*R* = 0.2, *P* = .16	r = −0.03, *P* = .84	r = −0.01, *P* = .95	*R* = 0.02, *P* = .92	*R* = 0.49, *P* < .01		*R* = 0.16, *P* = .54
	СОХ2	*R* = 0.11, *P* = .67	r = −0.49, *P* = .037	r = −0.02, *P* = .95	*R* = 0.15, *P* = .57	*R* = 0.45, *P* = .06	*R* = 0.16, *P* = .54	
EH	ER, epithelium	*R* = 0.49, *P* = .063	r = −0.16, *P* = .56	r = −0.17, *P* = .55	r = −0.11, *P* = 0.7		*R* = 0.44, *P* = .1	r = −0.18, *P* = .73
	ER, stroma	*R* = 0.1, *P* = .73	*R* = 0.02, *P* = .95	r = −0.04, *P* = .9	r = −0.08, *P* = .78	*R* = 0.44, *P* = .1		r = −0.47, *P* = .35
	СОХ2	*R* = 0.58, *P* = .23	r = −0.03, *P* = .95	*R* = 0.09, *P* = .86	*R* = 0.21, *P* = .69	r = −0.18, *P* = .73	r = −0.47, *P* = .35	
GEP	ER, epithelium	r = −0.25, *P* = .59	r = −0.04, *P* = .94	*R* = 0.32, *P* = .5	*R* = 0.21, *P* = .72		*R* = 0.56, *P* = .19	*R* = 0.62, *P* = .19
	ER, stroma	*R* = 0.2, *P* = .67	r = −0.62, *P* = .14	r = −0.2, *P* = .67	*R* = 0.18, *P* = .7	*R* = 0.56, *P* = .19		*R* = 0.63, *P* = .18
	СОХ2	*R* = 0.14, *P* = .79	r = −0.23, *P* = .65	*R* = 0.37, *P* = .47	*R* = 0.46, *P* = .36	*R* = 0.62, *P* = .19	*R* = 0.63, *P* = .18	
GFEP	ER, epithelium	*R* = 0.3, *P* = .12	r = −0.12, *P* = .53	*R* = 0.1, *P* = .62	*R* = 0.11, *P* = .59		*R* = 0.55, *P* < .01	*R* = 0.91, *P* = .013
	ER, stroma	*R* = 0.22, *P* = .25	*R* = 0.07, *P* = .72	*R* = 0.02, *P* = .93	*R* = 0.04, *P* = .85	*R* = 0.55, *P* < .01		*R* = 0.52, *P* = .29
	СОХ2	r = −0.34, *P* = .5	r = −0.85, *P* = .034	r = −0.31, *P* = .55	*R* = 0.12, *P* = .82	*R* = 0.91, *P* = .013	*R* = 0.52, *P* = .29	

There was a significant difference between patient groups, *P* < .05.

BMI = body mass index, EH = endometrial hyperplasia, EHP = endometrial hyperplastic process, ER = estrogen receptors, GEP = glandular endometrial polyps, GFEP = glandular-fibrous endometrial polyps.

## 5. Discussions

This study’s relevance is due to the high risk of malignant transformation and the problems related to menstrual cycle disorders, dysfunctional uterine bleeding, and anemia in women.^[8]^ Early detection of EHP is necessary for preventing endometrial carcinomas.^[24,25]^ ERs, which are estrogen targets, play a critical role in female genital pathophysiology and EHP development.^[26,27]^ Estrogen interacting with ERα induces endometrial proliferation during the proliferative phase of the menstrual cycle.^[9]^ To some extent, ER expression predicts the response to conservative treatment with progestins in women with EHP and stage I endometrial cancer.^[20]^ In addition, there is a constant need to optimize data on ER thresholds in normal and pathological tissues to prescribe hormone therapy correctly.^[28]^

COX2 is a crucial enzyme associated with inflammation and tumorigenesis.^[11,17,29]^ It regulates cell proliferation, differentiation, and apoptosis through several autocrine and paracrine transducers.^[29,30]^ COX2 expression may also be associated with inflammatory and dyshormonal disorders of genital organs.

We found that GEP occurred at a younger age than EH and GFEP. There was no association between EHP type and height, body weight, or BMI. Women with EHP tended to have increased BMI, with 51% either overweight or obese.

We detected ER expression in all the studied EHP types. Consistent with previous studies, it was high in the epithelial and stromal components in EH^[28,29]^ and EP^[31]^ samples.^[31–34]^ We also observed a predominance of ER expression in the epithelial component vs stromal component.^[35–43]^ It should be noted that all groups had cases of inhomogeneous immunostaining, consistent with a previous study. We found no association between ER expression in the epithelial and stromal components and EHP type. However, we discovered a direct correlation between ER expression in the endometrial glands’ epithelium and stroma.

COX2 was positively expressed in all tissue sample groups. Its expression was significantly higher than in intact tissues, indicating a potential role in the endometrium’s dyshormonal transformation.^[17]^ Unlike previous studies, all tissue samples with atypical EH had a positive signal in the endometrial glands’ epithelium.^[17,40,43]^ Consistent with some previous studies, EP tissue samples had high COX2 expression. However, these data contradict other studies, which reported lower COX2 expression in EP that did not differ significantly from samples with normal endometrium. Despite the data showing higher COX2 expression in EP than in EH.,^[17,30,33,40–43]^ we did not find any dependent COX2 expression on EHP subgroups.

We established a direct correlation between ER and COX2 expression in the endometrial epithelium. This finding might indicate their synergistic involvement in EHP initiation and progression and their possible role in subsequent tumor transformation. The proven involvement of COX2 in the progression of endometrial carcinomas^[40–43]^ can serve as a prognostic indicator for the EHP course and the risk of malignant transformation.

## 6. Conclusion

Hyperplastic processes of the endometrium are characterized by variability in ER and COX2 expression. Their expression levels do not depend on patients anthropometric parameters. Direct correlations were found between ER expression levels in the endometrial epithelium and stroma and ER and COX2 expression levels in the endometrial epithelium, potentially indicating their synergistic involvement in the initiation and progression of EHPs and their possible role in subsequent tumor transformation. The IHC results for ER and COX2 expression can serve as criteria for a differentiated approach to treatment strategy choice.

## Acknowledgements

This work was supported by the Ministry of Education and Science of Ukraine under Grant № 0121U100472 and Grant № 0123U100111; research theme of the Department of Pathology of Sumy State University under Grant № 0119U100887.

## Author contributions

**Conceptualization:** Nataliia Tsyndrenko, Mykola Lуndіn, Kateryna Sikora, Toufik Abdul-Rahman, Nataliia Hyriavenko, Anatolii Romaniuk.

**Data curation:** Nataliia Tsyndrenko, Mykola Lуndіn, Kateryna Sikora, Andrew Awuah Wireko, Toufik Abdul-Rahman, Nataliia Hyriavenko, Anatolii Romaniuk.

**Formal analysis:** Nataliia Tsyndrenko, Mykola Lуndіn, Kateryna Sikora, Andrew Awuah Wireko, Toufik Abdul-Rahman, Nataliia Hyriavenko, Anatolii Romaniuk.

**Funding acquisition:** Kateryna Sikora, Toufik Abdul-Rahman.

**Investigation:** Nataliia Tsyndrenko, Mykola Lуndіn, Kateryna Sikora, Andrew Awuah Wireko, Anatolii Romaniuk.

**Methodology:** Nataliia Tsyndrenko, Mykola Lуndіn, Kateryna Sikora, Toufik Abdul-Rahman, Nataliia Hyriavenko, Anatolii Romaniuk.

**Visualization:** Nataliia Hyriavenko.

**Writing – original draft:** Nataliia Tsyndrenko, Mykola Lуndіn, Kateryna Sikora, Andrew Awuah Wireko, Toufik Abdul-Rahman, Nataliia Hyriavenko, Anatolii Romaniuk

**Writing – review & editing:** Nataliia Tsyndrenko, Mykola Lуndіn, Kateryna Sikora, Andrew Awuah Wireko, Toufik Abdul-Rahman, Nataliia Hyriavenko, Anatolii Romaniuk
